# Fibroblast Growth Factor 18 Increases the Trophic Effects of Bone Marrow Mesenchymal Stem Cells on Chondrocytes Isolated from Late Stage Osteoarthritic Patients

**DOI:** 10.1155/2014/125683

**Published:** 2014-12-03

**Authors:** Zhenyu Zhang, Yan Wang, Mingchao Li, Jiaping Li, Jian Wu

**Affiliations:** ^1^Department of Orthopedic Surgery, The First Affiliated Hospital of Harbin Medical University, 23 You Zheng Street, Nangang District, Harbin 150001, China; ^2^Microscopic Hand Surgery, People's Hospital of Hainan Province, Haikou 570100, China

## Abstract

Coculture of mesenchymal stem cells with chondrocytes increases production of cartilaginous matrix. Chondrocytes isolated from late stage osteoarthritic patients usually lost their phenotype of producing cartilaginous matrix. Fibroblast growth factor 18 is believed to redifferentiate OA chondrocyte into functionally active chondrocytes. The aim of this study is to investigate the supportive effects of MSCs on OA chondrocytes and test if FGF18 could enhance the responsiveness of OA chondrocytes to the support of MSCs in a coculture system. Both pellet and transwell co-cultures were used. GAG quantification, hydroxyproline assay, and qPCR were performed. An ectopic models of cartilage formation was also applied. Our data indicated that, in pellets coculture of MSCs and OA chondrocytes, matrix production was increased in the presence of FGF18, comparing to the monoculture of chondrocytes. Results from transwell coculture study showed that expression of matrix producing genes in OA chondrocytes increased when cocultured with MSCs with FGF18 in culture medium, while hypertrophic genes were not changed by coculture. Finally, coimplantation of MSCs with OA chondrocytes produces more matrix than chondrocytes only. In conclusion, FGF18 can restore the responsiveness of OA chondrocytes to the trophic effects of MSCs. Coimplantation of MSCs and OA chondrocytes treated with FGF18 may be a good alternative cell source for regenerating cartilage tissue that is degraded during OA pathological changes.

## 1. Introduction

Osteoarthritis (OA) is known as the most common degenerative diseases in joints. Symptoms of OA include a group of mechanical abnormalities, which reflect the degradation of articular cartilage and the corresponding subchondral bones [1]. OA patients normally experience pain, tenderness, stiffness, locking, and/or effusion of joints. A lot of factors including genetics, developmental environment, metabolism, and mechanical injury are considered as causes for initiating degradation of cartilage. Once started, cartilage tissue will become thinner and thinner; then bony surfaces of joints will be protected and buffer less and less. Then subchondral bone may be damaged. As the most common type of arthritis, OA reduces the life experience of millions of patients in the United States and much more worldwide [2–4]. Current treatments mainly delay its progression. Most OA patients will eventually have to do surgery for total joint replacement.

Fibroblast growth factor 18 (FGF18) is identified as a new member of the fibroblast growth factor (FGF) family in late 1990s [5]. The gene encoding this protein was mapped to chromosome 5q34 [6]. Fgf18, together with* Fgf8* and* Fgf17,* is usually considered as one subfamily of FGFs. During development, endogenous Fgf18 is known to play an important role in skeletal growth as indicated by malformations of caldaria suture and growth plate in mice lacking FGF18 [7, 8]. Besides its stimulatory effects on chondrocyte proliferation and differentiation in the growth plate [9], Fgf18 was reported to be an anabolic factor on chondrocytes in articular cartilage [10]. It has also been reported that FGF18 may accelerate the biosynthesis of type II collagen synthesis and extracellular matrix deposition of chondrocytes [11]. Based on these reports as well as the fact that injection of rhFGF18 prevented cartilage degeneration in rat osteoarthritis models, FGF18 is believed to protect articular cartilage from intra-articular injury [12]. Furthermore, beneficial effects of FGF18 have also been shown in the repair of damaged cartilage in a rat study of injury-induced osteoarthritis, conducted by Moore et al. [13].

Trophic effects of mesenchymal stem cells (MSCs) are generally defined as an observation in which MSCs help other cells to survive, proliferate, and produce extracellular matrix by producing secreted factors into neighboring environment [14]. It is believed that MSCs may play a trophic role in many tissues. MSCs may improve the functions of the neural system by being injected into stroked brain of rats [15]. No MSCs were observed to differentiate into neurons or any other neuronal cells in the study. Similarly, MSCs can stimulate cardiomyocyte proliferation and vascular regeneration without differentiating into tissue-specific cells, both* in vitro* and* in vivo* [16, 17]. Recently, the trophic effects of MSCs in cartilage regeneration were demonstrated in coculture systems [18, 19]. MSCs are shown to increase extracellular matrix formation and proliferation of chondrocytes. Meanwhile, MSCs died overtime in the coculture with chondrocytes. Moreover, trophic effects were shown to be general phenomena which are observed in MSCs cultured in different medium and MSCs isolated from different tissue [20, 21]. Substitution of chondrocyte partially by MSCs may significantly reduce the number of chondrocytes needed to construct tissue engineering constructs (TE constructs) of cartilage.

In this study, we hypothesized that FGF18 could enhance the trophic effects of MSC when cocultured with chondrocytes derived from late stage OA patients. Both pellet and transwell coculture systems were used to study the interactions between MSCs and chondrocytes. An ectopic model of cartilage regeneration was applied to test the effects of FGF18* in vivo*.

## 2. Materials and Methods

### 2.1. Cell Culture and Expansion

The use of human material in this study has been approved by the local Medical Ethical Committee of Harbin Medical University. Severely damaged cartilage tissue was obtained from hip biopsies of patients with end stage osteoarthritis undergoing total hip replacement. Chondrocytes were isolated with previously published protocols [22]. Briefly, cartilage was dissected from underlying bone and connective tissue and was digested for 20–22 h in collagenase type II (0.15% Worthington) in DMEM supplemented with penicillin (100 U/mL) and streptomycin (100 mg/mL). Primary chondrocytes were then cultured in chondrocyte proliferation medium (DMEM supplemented with 10% FBS, 1 × nonessential amino acids, 0.2 mM Ascorbic acid 2-phosphate, 0.4 mM proline, 100 U/mL penicillin, and 100 *μ*g/mL streptomycin). Bone marrow biopsies were obtained from the same patients who underwent total hip replacement. Mesenchymal stem cells (MSCs) were derived from bone marrow as described previously [23]. Total bone marrow was plated at a density of 50 000 cells/cm^2^ in culture flasks in MSC proliferation medium (*α*-MEM, supplemented with 10% fetal bovine serum, 1% L-glutamine, 0.2 mM Ascorbic acid, 100 U/mL penicillin, 10 *μ*g/mL streptomycin, and 1 ng/mL bFGF), plus 1% heparin. Medium was refreshed every 3-4 days until confluence. Three donor pairs were used in this study. One pair means both cells types are from the same donor. All reagents used for cell culture were purchased from Invitrogen (Carlsbad, CA). Common chemicals were purchased from Sigma-Aldrich.

### 2.2. Coculture of MSCs and Chondrocytes in Pellets or Transwell

For pellet coculture, 100 000 chondrocytes and 100 000 MSC (ratio of 1 : 1) were seeded in one well of a round bottom ultralow attachment 96-well plate in serum-free medium with or without FGF18 (10 ng/mL) and centrifuged for 3 min at 500 ×g. Serum-free medium contains DMEM plus 1 × nonessential amino acids, 0.2 mM Ascorbic acid 2-phosphate, 0.4 mM proline, 100 U/mL penicillin, and 100 *μ*g/mL streptomycin. Medium was refreshed twice a week. Monoculture pellets of chondrocytes were made the same way with 200 000 cells.

For transwell coculture, 100 000 chondrocytes were seeded in one Millicell cell culture insert (0.45 *μ*m PCF, 12 mm Diameter) manufactured by Merk Millipore (Tullagreen, Ireland). Cell culture insert was then put in one well of 24-well plate where 100 000 MSCs were seeded. Serum-free medium with or without FGF18 (10 ng/mL) was used. For monoculture transwells, 100 000 chondrocytes were seeded in cell culture insert and one well of a 24-well plate, respectively. This is illustrated in Figure 2(a).

### 2.3. Histology

Cell pellets or TE constructs were fixed with 10% formalin overnight and embedded in Paraffin using routine procedures. Sections of 4 *μ*m were cut and stained for sulfated glycosaminoglycans (GAG) with Toluidine blue or Safranin O.

### 2.4. Quantitative GAG and DNA Assay

Cell pellets were washed with PBS and frozen overnight at −20°C. Subsequently, they were digested in 500 *μ*L digestion buffer 1 mg/mL proteinase K in Tris/EDTA buffer (pH7.6) for >16 h at 56°C. Glycosaminoglycan (GAG) content was spectrophotometrically determined with 1,9-dimethylmethylene blue chloride (DMMB) staining in PBE buffer (14.2 g/L Na_2_HPO_4_ and 3.72 g/L Na_2_EDTA, pH 6.5) using an ELISA reader (TECAN, Grodig, Austria) at an absorbance of 520 nm with chondroitin sulfate as a standard. Cell number was determined by quantification of total DNA using a CyQuant DNA Kit (Molecular Probes, Eugene, OR), according to the manufacturer's instructions.

Three pellets of each donor pair were used for GAG and DNA assay to show technical variations.

### 2.5. Hydroxyproline Assay

Collagen contents of the aggregate were assessed by the hydroxyproline assay, using previously described method [24]. Briefly, cell aggregates were digested in papain buffer (0.5 mg/mL of papain dissolved in 0.1 M Na_2_HPO_4_, 5 mM EDTA, 5 mM L-cysteine HCl) overnight. The digested solutes were hydrolyzed in 6 N HCl at 110°C overnight. Hydroxyproline was then assayed spectrophotometrically at 560 nm after reaction with 0.05 M of chloramine-T and 10% (w/v in 2-methoxyethanol) *ρ*-dimethylaminobenzaldehyde. A standard curve was generated with L-hydroxyproline for calculating the hydroxyproline concentration.

### 2.6. COL II Enzyme-Linked Immunosorbent Assay (ELISA)

Cell pellets and TE constructs were digested in a mixture of enzymes containing 0.5 mg/mL of hyaluronidase and 0.25 mg/mL of Pepsin A for more than 16 hours. Quantitative analysis of COL II deposition in cell pellets as well as TE constructs was then carried out using an ELISA kit purchased from Chondrex Inc. (Redmond, WA) following manufacturer's protocol.

### 2.7. RNA Isolation and Quantitative PCR

Total RNA samples of chondrocytes seeded in cell culture inserts were isolated with the QIAamp DNA Mini Kit (Qiagen, Hilden, Germany). One microgram of total RNA was reverse-transcribed into cDNA using the iScript cDNA Synthesis kit (Bio-Rad, Hercules, CA). qPCR was performed on cDNA samples by using the iQ SYBR Green Supermix (Bio-Rad, Hercules, CA). PCR reactions were carried out on MyiQ2 Two-Color Real-Time PCR Detection System (Bio-Rad, Hercules, CA) under the following conditions: cDNA was preheated for 15 min at 96°C, denatured for 5 min at 95°C, and followed by 45 cycles, which consist 15 s at 95°C, 15 s at 60°C, and 30 s at 72°C in one cycle. For each reaction a melting curve was generated to test primer dimer formation and nonspecific priming. The primers for real-time PCR are listed in Supplementary Table 1  (see Supplementary Material available online at http://dx.doi.org/10.1155/2014/125683). Calculation of relative expression was performed with Bio-Rad iQ5 optical system software (version 2.0) using the double delta Ct method [25]. GAPDH was used for normalization.

### 2.8. Ectopic Cartilage Formation in Alginate Gel Implanted in Nude Mice

MSCs and chondrocytes were mixed with a ratio of 1 : 1 and then resuspended in 2% alginate in PBS at a density of 1 × 10^7^ cells/mL. Constructs were made by transferring 100 *μ*L of alginate cell suspension to 100 mM CaCl_2_ solution and gelifying for 5 minutes at 37°C. Constructs were washed with PBS and then DMEM. Constructs with chondrocytes only were served as control. Constructs were cultured in serum-free medium with or without FGF18 (10 ng/mL) for 2 weeks before being implanted into nude mice subcutaneously. For each condition, 4 constructs were made to represent technical variations. Before the surgery, twelve 6-week-old male BALB/C nude mice (animal center of Harbin Medical University) were anesthetized. Then, four subcutaneous pockets were made on the back of a mouse. One construct was put in one pocket. The locations of the constructs were randomized and recorded. Eight weeks after implantation, mice were sacrificed and implanted constructs were then carefully separated from surrounding fibrous capsule and washed in PBS for histological analysis and quantitative GAG analysis.

### 2.9. Statistical Analysis

All statistical analyses were made by using Student's *t*-test for paired samples. *P* values of <0.05 were considered as statistically significant.

## 3. Results

### 3.1. Coculture of MSCs and OA Chondrocytes Increases Matrix Production in the Presence of FGF18

**Figure 1:**
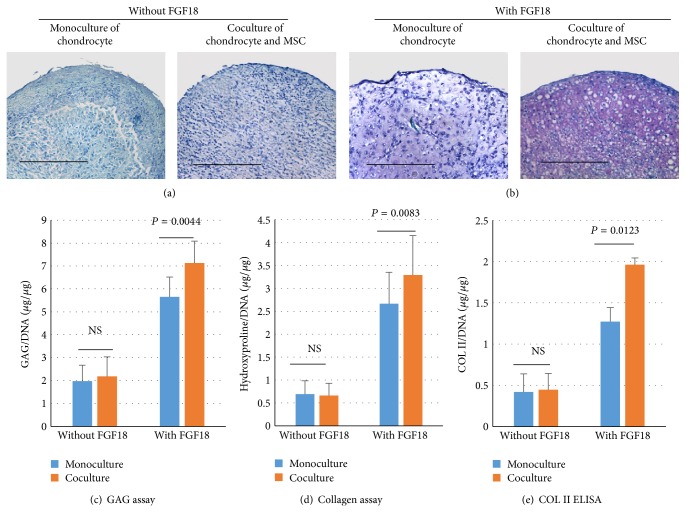
Coculture of MSCs and OA chondrocytes increases GAG formation and collagen biosynthesis with the presence of FGF18. (a) Toluidine blue staining was performed at week 4 after aggregation that was cultured in medium without FGF18 to detect GAGs. Scale bar = 100 *μ*m. (b) Toluidine blue staining was performed at week 4 after aggregation that was cultured in medium containing FGF18 to detect GAGs. Scale bar = 100 *μ*m. (c) GAG assay was performed to quantify GAG deposited in cell pellets (*n* = 3) at week 4 after aggregation. Total GAGs (*μ*g) were normalized to total DNA (*μ*g). Error bar reflects standard deviation. *P* values were calculated with Student's *t*-test. (d) Hydroxyproline assay was carried out to measure total collagen contents (*n* = 3). Total collagen (*μ*g) was normalized to total DNA (*μ*g). Error bar reflects standard deviation. *P* values were calculated with Student's *t*-test. (e) Deposition of COL II in pellets was measured by ELISA (*n* = 3). COL II contents were normalized to total DNA. *P* values were calculated with Student's *t*-test.

Four weeks after aggregation, cell pellets of monoculture and coculture were harvested for histological examination, GAG assay, and collagen quantification. As shown in Figure 1(a), not much GAG was deposited into extracellular matrix in pellets cultured in serum-free medium without FGF18. This is not surprising because chondrocytes isolated from end stage OA patients already lost their ability to produce cartilaginous matrix. With or without the supports from MSCs, they cannot lay down matrix rich in GAG and collagen. Upon stimulation of FGF18, these OA chondrocytes quickly regain their machinery for matrix synthesis and deposit abundant GAG in the pellets (Figure 1(b)). Building on this redifferentiation process, MSCs could further promote GAG synthesis of chondrocytes due to their trophic effects. Quantitative measurements for GAG and total collagen confirmed our impression on histological staining (Figures 1(c) and 1(d)). Without FGF18 in the medium, 1 *μ*g of DNA is roughly corresponding to 2 *μ*g of GAG and 1 *μ*g hydroxyproline. And there is no difference between monoculture and coculture pellets. Once FGF18 is added to the medium, three- to fourfold increases were seen in GAG and collagen quantification. More importantly, GAG produced in coculture pellets was significantly more than that in monoculture pellets. The same trend was observed in collagen quantification. Since hydroxyproline assay cannot distinguish cartilage specific collagen type II (COL II) from other collagens, ELISA was performed to quantify the amount of COL II in cell pellets. Upon stimulation of FGF18, monoculture and coculture pellets deposit 3-4 times more COL II in matrix. As expected, coculture pellets contain significantly more COL II than monoculture pellets (Figure 1(e)). This indicated that trophic effects of MSCs may increase matrix formation of OA chondrocytes with the redifferentiation of chondrocytes stimulated by FGF1.

### 3.2. Transwell Coculture Increases Expression of Matrix Producing Genes in OA Chondrocytes in the Presence of FGF18

**Figure 2:**
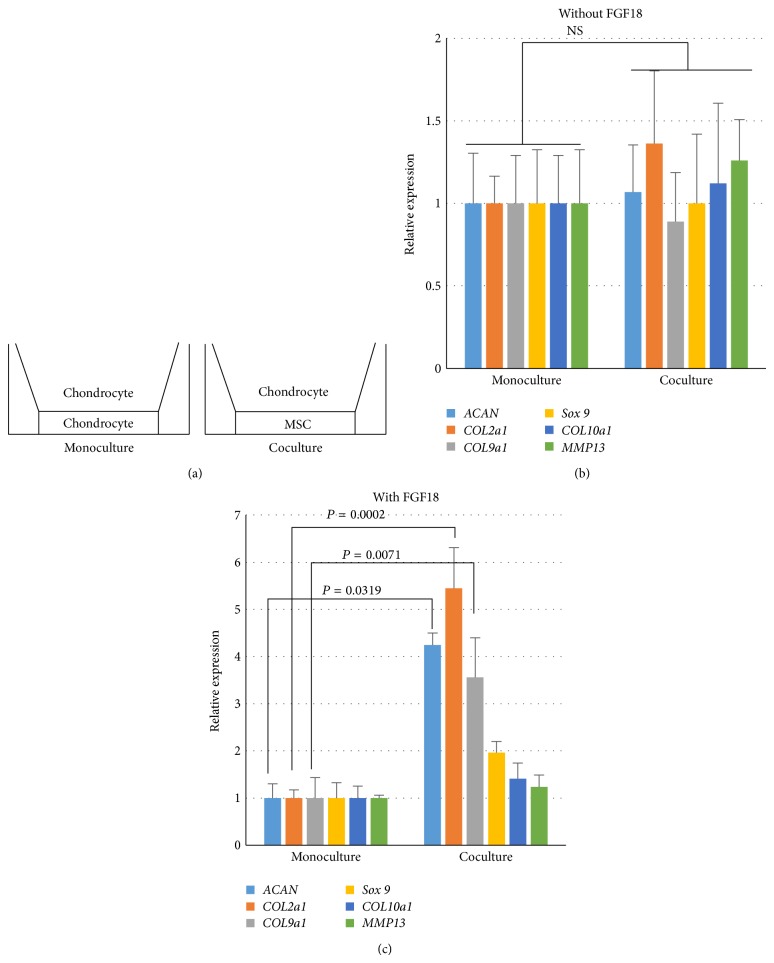
Expression of chondrogenic genes was measured by qPCR in OA chondrocytes cultured in transwells. (a) Diagram shows how monoculture or coculture is performed in transwell systems. (b) Expression of chondrogenic genes in chondrocytes cultured in transwells in medium without FGF18. Beta-actin was used for normalization. Monoculture was chosen as reference. Number in coculture represents the relative expression level of corresponding gene comparing to monoculture. Three donor pairs were analyzed. NS: nonsignificance. (c) Expression of chondrogenic genes in chondrocytes cultured in transwells in medium containing FGF18 (10 ng/mL). Beta-actin was used for normalization. Monoculture was chosen as reference. Number in coculture represents the relative expression level of corresponding gene comparing to monoculture. Three donor pairs were analyzed. *P* values are calculated with Student's *t*-test.

To specify the influence of MSCs on chondrocytes with the presence of FGF18, a transwell coculture system was used (Figure 2(a)). Real-time PCR was performed to quantify the expression of chondrogenic genes in chondrocytes. Without FGF18 in the medium, coculturing with MSC could not increase expression of chondrogenic genes in chondrocytes (Figure 2(b)). However, MSCs significantly increases gene expressions in chondrocytes with the presence of FGF18 (Figure 2(c)). In particular, chondrocytes expressed 5 times more COL2a1 in coculture with MSC than in monoculture.

### 3.3. Coimplantation of MSCs with OA Chondrocytes Produces More Matrix Than Chondrocytes Only

**Figure 3:**
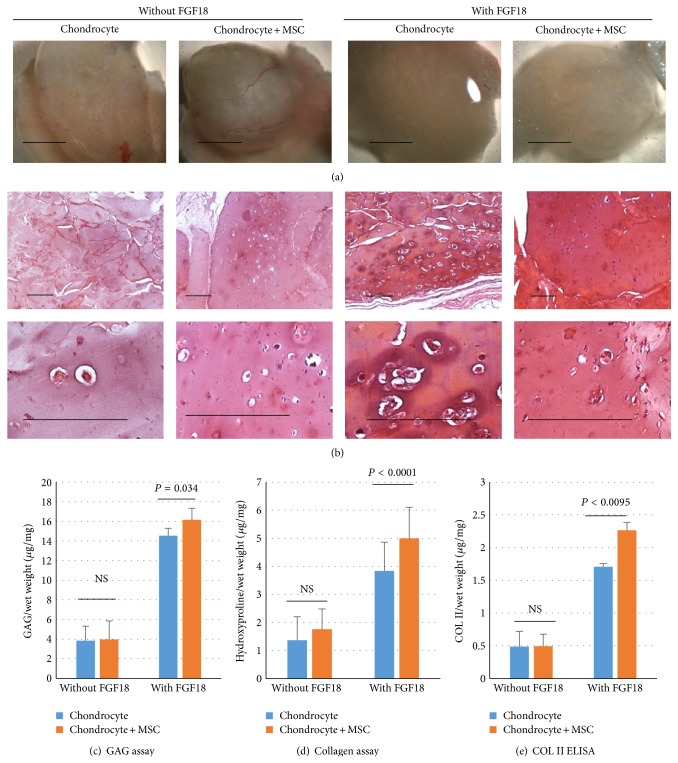
Coimplantation of MSCs and chondrocyte in alginate hydrogel subcutaneously in nude mice. (a) Overview of tissue engineering constructs containing chondrocytes only or mixture of MSC and chondrocyte. Hydrogel was loaded with or without FGF18 (10 ng/mL). Bar = 5 mm. (b) Tissue engineering constructs were sectioned and stained with Safranin O. Lower panel is the enlarged pictures of the inserts in upper panel. Bar = 50 *μ*m. (c) GAGs were quantified in tissue engineering constructs GAG assay (*n* = 3). Total GAGs (*μ*g) were normalized to wet weight (mg). Data was presented as mean ± standard deviation. *P* values were calculated with Student's *t*-test. NS: nonsignificance. (d) Collagen contents were measured in tissue engineering constructs by hydroxyproline assay (*n* = 3). Total collagen (*μ*g) was normalized to wet weight (mg). Data was presented as mean ± standard deviation. *P* values were calculated with Student's *t*-test. NS: nonsignificance. (e) Collagen type II deposition was tested in tissue engineering constructs by ELISA (*n* = 3). Total COL II (*μ*g) was normalized to wet weight (mg). Data was presented as mean ± standard deviation. *P* values were calculated with Student's *t*-test. NS: nonsignificance.

To evaluate previous findings* in vivo*, chondrocytes and MSCs (ratio 1 : 1) were coimplanted into subcutaneously immune-deficient mice with alginate as carrying vehicle. Before implantation, TE constructs were cultured in serum-free medium with or without FGF18. Then, eight weeks after implantation, histological examination as well as GAG and collagen quantification was performed. Figure 1(a) shows the gross-looking of TE constructs under stereomicroscope. In general, TE constructs of all groups look similar. Those ones not cultured in FGF18 seem to have more angiogenesis. As shown in Figure 3(b), there was some accumulation of ECM components in the pericellular regions in all experimental groups. However, TE constructs cultured in FGF18 obviously deposited more GAGs than constructs cultured without FG18. Quantification of GAG and collagen confirmed that coimplantation after FGF18 culture group produced most GAGs and collagens when comparing with other experimental groups (Figures 3(c) and 3(d)). Then, ELISA was used to quantify cartilage specific COL II in TE constructs. Comparing the groups cultured with or without FGF18, at least threefold increase was observed in COL II production between two groups (Figure 3(e)). Again, coimplantation TE constructs contain significantly more COL II than chondrocyte TE constructs.

## 4. Discussion

In this study, influence of FGF18 on the trophic effects of MSC was studied in coculture system of MSCs and chondrocytes derived from late stage OA patients. Pellet coculture system showed that MSCs increased GAG formation and collagen synthesis of chondrocytes with FGF18 in culture medium. Data from transwell coculture systems indicated that MSCs promoted the expressions of chondrogenic genes in chondrocytes in the presence of FGF18. Ectopic formation confirmed that FGF18 treatment restored the responsiveness of OA chondrocyte to MSCs* in vivo*.

In previous studies, it was shown in pellet coculture of chondrocytes and bone marrow derived MSCs that the beneficial effects of coculture on cartilage matrix formation are largely due to the support of MSCs on chondrocyte proliferation and matrix secretion [18, 19]. In these pellet cocultures, cartilage matrix was mainly produced by chondrocytes; however, MSCs were not actively undergoing chondrogenic differentiation. Another report then showed that the trophic effects of MSCs on chondrocytes are independent of culture conditions and were found in cocultures of chondrocytes with various sources of MSCs [17]. Moreover, Meretoja et al. reported that MSCs can also support matrix production of chondrocytes through trophic effects in a setup of coculture system in hydrogels [26]. These studies indicate that the most promising cell source for cartilage engineering was cocultures, as they have a potential to decrease the need for primary chondrocyte harvest and expansion while obtaining a stable highly chondrogenic phenotype.

Chondrocytes isolated from late stage OA patients usually lost their capability to deposit cartilaginous matrix. As indicated in this study, the coculture pellets of MSCs and OA chondrocytes did not deposit sufficient amount of GAGs and collagens. It is reported that transforming growth factor-*β*3 (TGF-*β*3) could induce cartilaginous matrix formation in cocultures of OA chondrocytes and MSC in both pellets and biodegradable scaffolds [20, 27]. TGF-*β*3 could also redifferentiate chondrocytes that lost their phenotype during* in vitro* expansion in coculture system on porous scaffolds [28]. Using TGF-*β* to redifferentiate OA chondrocytes may raise concerns of osteoarthritis [29]. It is reported that high concentrations of active TGF-*β*1 in subchondral bone may initiate osteoarthritis in knee joints, and inhibition of TGF-*β* signaling could attenuate the pathological process of osteoarthritis [30]. Instead of TGF-*β*, we used FGF18 in this study to restore phenotype of OA chondrocytes. Our data indicated that FGF18 may increase the responsiveness of OA chondrocytes to the trophic effects of MSCs. Besides improving GAG production which had been reported in previous studies of coculture, this study provided more detailed results on collagen deposition especially COL II synthesis. This is the first evidence that MSCs stimulate chondrocytes to produce more COL II synthesis to extracellular matrix in a coculture or coimplantation system.

Taken together, results from this study demonstrate that FGF18 in culture medium made OA chondrocytes respond to the supportive effects of MSCs. In pellet coculture system as well as ectopic coimplantation, MSCs promote GAGs and COL II production of OA chondrocytes with the presence of FGF18. Coimplantation of MSCs and OA chondrocytes may be a useful source for regenerating cartilage tissue from OA chondrocytes.

## Supplementary Material

The primers for real-time PCR are obtained from Harvard primer bank (http://pga.mgh.harvard.edu/primerbank/). The primers which produce the shortest amplicons are usually picked. Size for each primer pair was also checked by resolving PCR products on agarose gel.
