# Supplementary material for: Fibroblast Growth Factor 18 Increases the Trophic Effects of Bone Marrow Mesenchymal Stem Cells on Chondrocytes Isolated from Late Stage Osteoarthritic Patients
Source: Stem Cells Int. 2014 Dec 3;2014:125683. doi: 10.1155/2014/125683 (PMC4269084; doi:10.1155/2014/125683)
Supplement: Supplementary file 1 — The primers for real-time PCR are obtained from Harvard primer bank (http://pga.mgh.harvard.edu/primerbank/). The primers which produce the shortest amplicons are usually picked. Size for each primer pair was also checked by resolving PCR products on agarose gel. [file 125683.f1.docx]

Supplementary table 1 Sequences of primers used for qPCR. Bp=base pairs.

| **Gene Name** | **Primer Sequence** | **Product size (bp)** | **GenBank Accession** |
| --- | --- | --- | --- |
| GAPDH | Forward: 5’ ACAACTTTGGTATCGTGGAAGG 3’  Reverse: 5’ GCCATCACGCCACAGTTTC’ | **101** | **NM_001256799** |
| ACAN | Forward: 5’CCCCTGCTATTTCATCGACCC 3’  Reverse: 5’GACACACGGCTCCACTTGAT 3’ | **90** | **NM_013227** |
| Sox9 | Forward: AGCGAACGCACATCAAGAC 3’  Reverse: 5’ CTGTAGGCGATCTGTTGGGG 3’ | **85** | **NM_000346** |
| COL 9a1 | Forward: GGCAGTAGAGGAGAATTAGGACC 3’  Reverse: 5’ GTTCACCGACTACACCCCTG 3’ | **142** | **NM_078485** |
| COL2a1 | Forward: 5’ CCAGATGACCTTCCTACGCC 3’  Reverse: 5’ TTCAGGGCAGTGTACGTGAAC 3’ | **186** | **NM_001844** |
| COL 10a1 | Forward: 5’ATGCTGCCACAAATACCCTTT 3’  Reverse: 5’ GGTAGTGGGCCTTTTATGCCT 3’ | **107** | **NM_000493** |
| MMP13 | Forward: 5’ACTGAGAGGCTCCGAGAAATG 3’  Reverse: 5’ GAACCCCGCATCTTGGCTT 3’ | **103** | **NM_002427** |
